# A Preliminary Study of the Heating Effect of the Tibetan Plateau

**DOI:** 10.1371/journal.pone.0068750

**Published:** 2013-07-31

**Authors:** Yonghui Yao, Baiping Zhang

**Affiliations:** State Key Laboratory of Resource and Environmental Information System, Institute of Geographic Sciences and Natural Resources Research, Chinese Academy of Sciences, Beijing, China; The Ohio State University, United States of America

## Abstract

The immense and towering Tibetan Plateau acts as a heating source and, thus, deeply shapes the climate of the Eurasian continent and even the whole world. However, due to the scarcity of meteorological observation stations and very limited climatic data, little is quantitatively known about the heating effect of the plateau and its implications. This paper firstly collects climate data (2001–2007) from 109 observation stations and MODIS-based estimated monthly mean temperature data in the plateau and the neighboring Sichuan Basin, and conducts correlation and simple linear regression to reveal the altitudinal pattern of temperature. Then, according to the linear relationships of temperature and altitude for each month, it compares air temperature differences on the same elevation between the main plateau and surrounding mountains and the Sichuan Basin so as to quantify the heating effect and discuss its implication on timberline of the plateau. The results show that: 1) the heating effect of the plateau is significant. The temperature of the main plateau area was higher than that of free air on the same elevation above the neighboring areas; on the elevation of 4500 m (the main plateau), temperature is 1–6°C higher in the main Plateau than over the Sichuan Basin for different months and 5.9–10.7°C higher than in the Qilian Mountains in the northeastern corner of the plateau. 2) Even at altitudes of 5000–6000 m in the main Plateau, there are 4 months with a mean temperature above 0°C. The mean temperature of the warmest month (July) can reach 10°C at about 4600–4700 m. This may help explain why the highest timberline in the northern hemisphere is on the southeastern Tibetan Plateau.

## Introduction

One of the most significances of the existence of the immense and towering Tibetan Plateau is that it thermodynamically shapes the climate of the Eurasian continent and even the whole world, and thus its geographical and ecological patterns are very special in the world. Previous researches have revealed that the highest timberline in the northern hemisphere is found on the south-eastern Tibetan Plateau [1]–[4], at about 4600–4700 m [3], [4] even higher (4900 m) on sunny slopes [2]. Comparatively, the alpine timberline is usually on elevation of 2500–3000 m at similar latitudes in other areas. Many contributed it to the so-called “Mass elevation effect” of the Tibetan Plateau [5]–[7]. Tollner (1949) stated that large mountain massifs have a positive effect on the altitudinal position of the timberline [8] because larger mountain massifs serve as a heating surface absorbing solar radiation and transforming it to long-wave energy; Consequently, temperature is higher than in the free atmosphere at any given elevation [7]. Many reports about Alps also stated that longer and warmer growing season at any given elevation than in the outer mountain ranges make timberline rise for about 400 m higher in the central Alps compared to the outer ranges [7], [9], [10]. The strong heating effect of elevated mountains should be responsible for timberline rising in central mountains.

The massive Tibetan Plateau has been found to be a heat source in summer (March through September) [11]–[15]. This has been recognized by many scientists since the 1950s [11]–[20]. Flohn (1957, 1968) proposed that the plateau surface is warmer in summer than adjacent free air, as a result of the altitudinal increase in solar radiation and relative constancy with height of effective infrared radiation [21], [22]. Barry (2008) suggested that two factors contribute to the heating effect in the mountain atmosphere: sensible heat transfer from the surface and the latent heat of condensation owing to precipitation from orographically induced cumulus development [23]. Chinese scientists have already carried out many studies on effects of this heat source on atmospheric general circulation [12], [14], [15], [24]–[26]. Calculations by Yeh (1982) indicated a total daily energy transfer from the plateau to the atmosphere of 231 W m^−2^ in June [12]. The maximum heating rates in June for the layer between 600 mb and 150 mb are +1.8°C day^−1^ from sensible heat and +1.4°C day^−1^ from latent heat; along with a radiative cooling of −1.5°C day^−1^, this gives a net heating of +1.7°C day^−1^ [12]. Various estimates suggest that the heating is about 2°C day^−1^ over the eastern half of the plateau [11]. Such a significant heating effect must impact ecological and geographical patterns on the plateau. Zheng et al. (1990) and Liu et al. (2003) mentioned that the plateau heating effect is important to the spatial pattern of mountain altitude belts [4], [27]. However, due to the scarcity of meteorological observation stations and very limited climatic data, previous works on the heating effect have focused on heat exchange between the atmosphere over the plateau and surrounding areas; little is quantitatively known about heating effect of the plateau and its forcing on plateau geo-ecological patterns. For instance, although many studies and field surveys have proposed the central mountains are warmer than the outer ranges at given elevations, we still do not know how much the main Plateau is warmer than the surrounding areas. Thus, this paper tries to quantify the plateau heating effect by comparing the temperature difference between the plateau and adjacent lowlands at same elevation, and to discuss why the highest timberline is on the Tibetan Plateau from the point of the changes of temperature with altitude in the plateau.

## Sites and Methods

**Figure 1:**
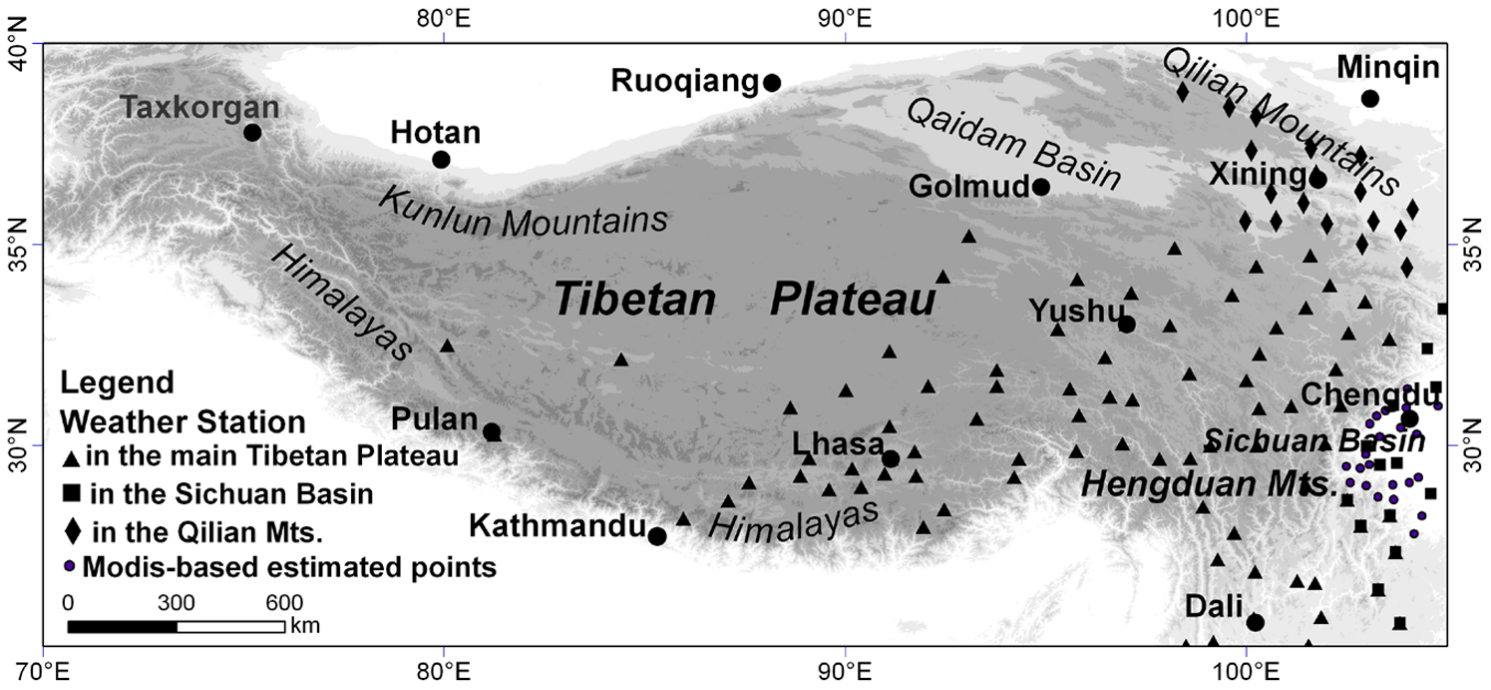
Study area and map of weather station distribution.

The study area stretches from latitudes 25–40°N and longitudes 70–105°E (Figure 1), and includes the entire Tibetan Plateau and adjacent areas. The plateau covers an area of nearly 2.5 million km^2^, and most parts of the plateau lies between 4000 m and 6000 m. The Himalayas, Hengduan Mountains and Kunlun Mountains, mostly 6000–7500 m above sea level, are situated at the southern, eastern and northern edges of the plateau, respectively. They and the broad high plateau between them constitute the main body of the plateau. The Qaidam Basin in the northeast is only about 3000 m and separates the Qilian Mountains from the main plateau. We selected the Sichuan Basin to the east of the plateau as the area from comparison. It is lower than 1000 m, and its western mountains are mostly lower than 3000 m.

Monthly mean temperature data in the study area are from 109 meteorological stations for 2001–2007, downloaded from the China Meteorological Information Center (http://cdc.cma.gov.cn/index.jsp). The stations were divided into three groups: 1) the main plateau group: 83 stations at elevations from 1109 m to 5336 m; 2) the Qilian Mountains group: 18 stations at elevations from 1826 m to 3400 m; and 3) the Sichuan Basin group: 7 stations from 332 m to 2990 m. For absent of high-elevation stations in Sichuan group (there is only one station above 1000 m), another 22 random points acquired from MODIS-based estimated temperature data which retrieved from MODIS Land Surface Temperature data combining with 137 meteorological data for 2001–2007, ASTER GDEM data by Geographical Weighted Regression methods. The RMSE for the estimation is 1.13–1.53°C [28].

Then, in order to reveal the altitudinal pattern of monthly mean temperature, correlation and linear regression analysis are conducted. Firstly, correlation analysis is conducted to test relationship and the significance of temperature with altitude. Then linear regression models of monthly temperature with altitude are constructed for the three aforementioned groups as follows:
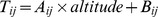
Where 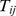 is air temperature (°C) modeled by the equation; Altitude equals elevation above sea level (m); i represents the three groups; j is month (from January through December). For each equation, two coefficients were computed: 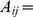
regional average lapse rate (°C/m), and 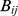 = temperature at sea level (0 m; °C).

Based on the equations of temperature with altitude by month (j) in the three groups, temperature differences (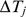) between the main plateau, Qilian mountains and Sichuan Basin were calculated at given elevations for each month. If 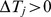, then the main plateau temperature was higher than free air temperature in the adjacent area, at the given elevation. According to those 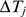 greater than 0°C, the values and sustained period of the heating effect are quantified.

## Results

### Altitudinal pattern of monthly mean temperature in the main Plateau

#### Correlation of monthly mean temperature with elevation

**Figure 2:**
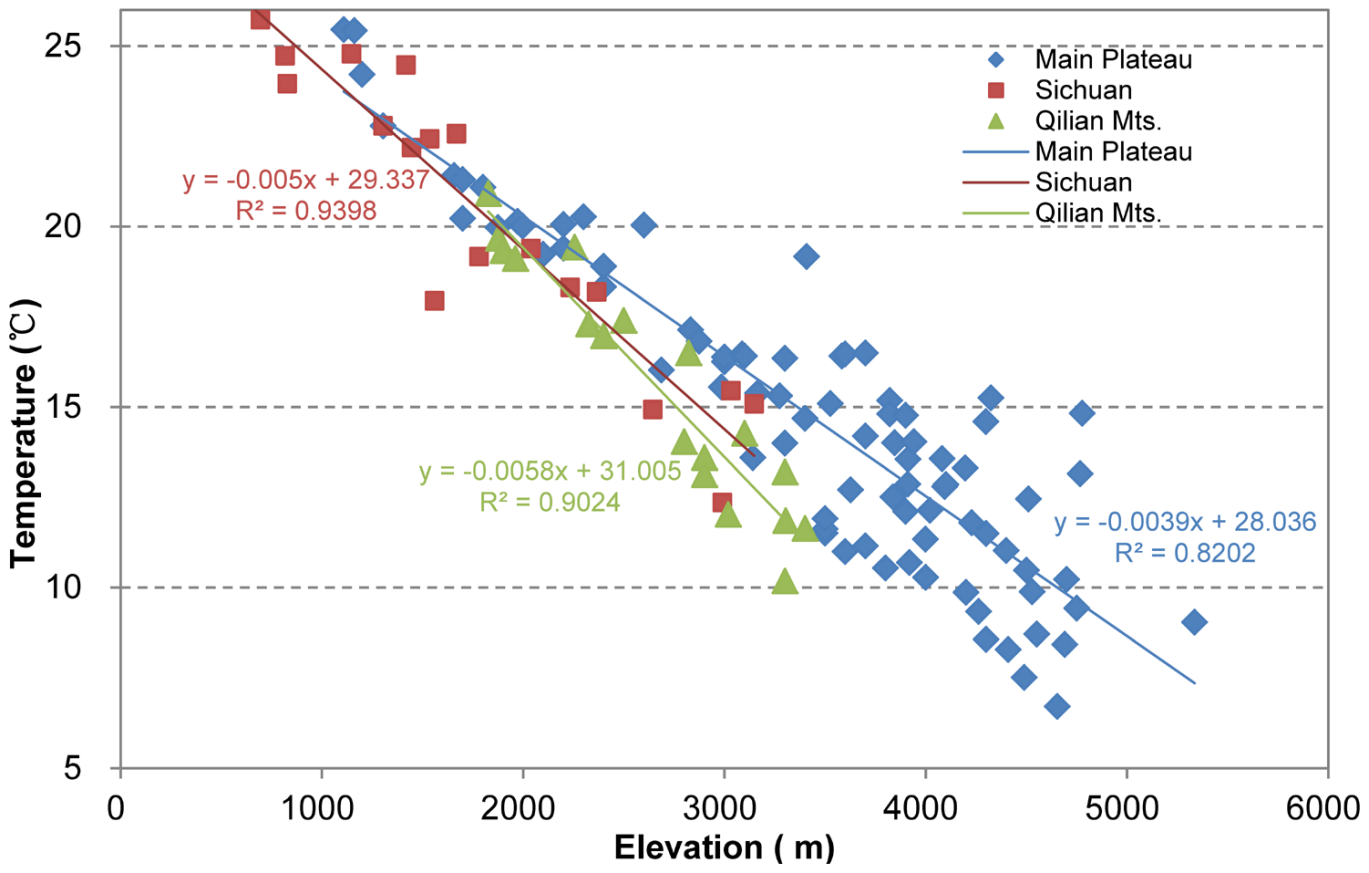
Changes of monthly mean temperature with elevation in July.

Correlation analysis indicates that, temperature is significantly negatively correlated with elevation and it is significant at the 0.01 level (Table 1). Determination coefficients (R^2^) and F-Prob value indicate that the linear regression model could be adequately used to describe the changes of temperature with elevation (Table 1). Monthly mean temperatures are decreasing with altitude increasing in each month for the three regions (Table 2, Figure 2), but there are regional difference and seasonal difference. Firstly, for every region, the regional average lapse rate (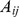) is different in season. It is lower in summer and winter and higher in spring and autumn (Table 2). Secondly, for every month, the regional average lapse rate is different in region. It is lower for Sichuan Basin and higher for the main Plateau and Qilian Mountains in spring and winter, but it is lowest for the main Plateau in summer (Table 2).

**Table 1:** The correlation and linear regression of monthly mean Temperature with altitude.

Region	Analysis	Parameters	Jan.	Feb.	Mar.	Apr.	May	Jun.	Jul.	Aug.	Sept.	Oct.	Nov.	Dec.
Main Plateau	Correlation	Pearson Correlation	−0.820	−0.870	−0.890	−0.921	−0.901	−0.871	−0.907	−0.909	−0.885	−0.888	−0.88	−0.834
		Sig. (2-tailed)	0.000	0.000	0.000	0.000	0.000	0.000	0.000	0.000	0.000	0.000	0.000	0.000
	Linear Regression	R^2^	0.67	0.76	0.79	0.85	0.81	0.76	0.82	0.83	0.78	0.79	0.77	0.70
		F-Prob	0.000	0.000	0.000	0.000	0.000	0.000	0.000	0.000	0.000	0.000	0.000	0.000
Qilian Mountains	Correlation	Pearson Correlation	−0.872	−0.894	−0.895	−0.916	−0.948	−0.96	−0.950	−0.94	−0.93	−0.934	−0.91	−0.836
		Sig. (2-tailed)	0.000	0.000	0.000	0.000	0.000	0.000	0.000	0.000	0.000	0.000	0.000	0.000
	Linear Regression	R^2^	0.76	0.80	0.80	0.84	0.90	0.92	0.90	0.88	0.86	0.87	0.83	0.70
		F-Prob	0.000	0.000	0.000	0.000	0.000	0.000	0.000	0.000	0.000	0.000	0.000	0.000
Sichuan	Correlation	Pearson Correlation	−0.942	−0.93	−0.954	−0.956	−0.972	−0.971	−0.969	−0.965	−0.97	−0.959	−0.953	−0.932
		Sig. (2-tailed)	0.000	0.000	0.000	0.000	0.000	0.000	0.000	0.000	0.000	0.000	0.000	0.000
	Linear Regression	R^2^	0.87	0.86	0.91	0.91	0.95	0.94	0.94	0.93	0.94	0.92	0.91	0.87
		F-Prob	0.000	0.000	0.000	0.000	0.000	0.000	0.000	0.000	0.000	0.000	0.000	0.000

Note: Correlation is significant at the 0.01 level (2-tailed).

**Table 2:** Linear regression models for the three regions in each month.

Month	The main Plateau	The Sichuan Basin	The Qilian Mountains
Jan.	*y* = −0.0057*x*+16.591 *R* ^2^ = 0.6696	*y* = −0.0043*x*+8.866 *R* ^2^ = 0.8867	*y* = −0.0057*x*+6.1174 *R* ^2^ = 0.7599
Feb.	*y* = −0.0061*x*+20.489 *R* ^2^ = 0.7573	*y* = −0.0045*x*+12.325 *R* ^2^ = 0.8647	*y* = −0.0061*x*+11.507 *R* ^2^ = 0.7988
Mar.	*y* = −0.0061*x*+23.975 *R* ^2^ = 0.7913	*y* = −0.0051*x*+16.788 *R* ^2^ = 0.9099	*y* = −0.0065*x*+17.107 *R* ^2^ = 0.8009
Apr.	*y* = −0.0058*x*+26.807 *R* ^2^ = 0.8492	*y* = −0.0056*x*+21.81 *R* ^2^ = 0.9141	*y* = −0.0061*x*+22.506 *R* ^2^ = 0.8399
May	*y* = −0.0051*x*+27.588 *R* ^2^ = 0.8124	*y* = −0.0059*x*+25.641 *R* ^2^ = 0.9456	*y* = −0.0064*x*+26.663 *R* ^2^ = 0.8989
Jun.	*y* = −0.0043*x*+28.101 *R* ^2^ = 0.7585	*y* = −0.0052*x*+27.543 *R* ^2^ = 0.9424	*y* = −0.0062*x*+29.933 *R* ^2^ = 0.9221
Jul.	*y* = −0.0039*x*+28.306 *R* ^2^ = 0.8202	*y* = −0.0050*x*+29.337 *R* ^2^ = 0.9398	*y* = −0.0058*x*+31.005 *R* ^2^ = 0.9024
Aug.	*y* = −0.0039*x*+27.698 *R* ^2^ = 0.8261	*y* = −0.0048*x*+28.299 *R* ^2^ = 0.9305	*y* = −0.0058*x*+30.255 *R* ^2^ = 0.8838
Sept.	*y* = −0.004*x*+25.704 *R* ^2^ = 0.7835	*y* = −0.0047*x*+24.851 *R* ^2^ = 0.9408	*y* = −0.0053*x*+24.545 *R* ^2^ = 0.8649
Oct.	*y* = −0.0051*x*+24.369 *R* ^2^ = 0.7879	*y* = −0.0052*x*+20.837 *R* ^2^ = 0.9201	*y* = −0.0058*x*+19.909 *R* ^2^ = 0.8714
Nov.	*y* = −0.0058*x*+21.259 *R* ^2^ = 0.7744	*y* = −0.0051*x*+16.28 *R* ^2^ = 0.9084	*y* = −0.0063*x*+14.246 *R* ^2^ = 0.8274
Dec.	*y* = −0.0055*x*+16.85 *R* ^2^ = 0.6952	*y* = −0.0045*x*+10.326 *R* ^2^ = 0.8678	*y* = −0.0057*x*+7.0039 *R* ^2^ = 0.6987

#### Altitudinal pattern of monthly mean temperature

**Figure 3:**
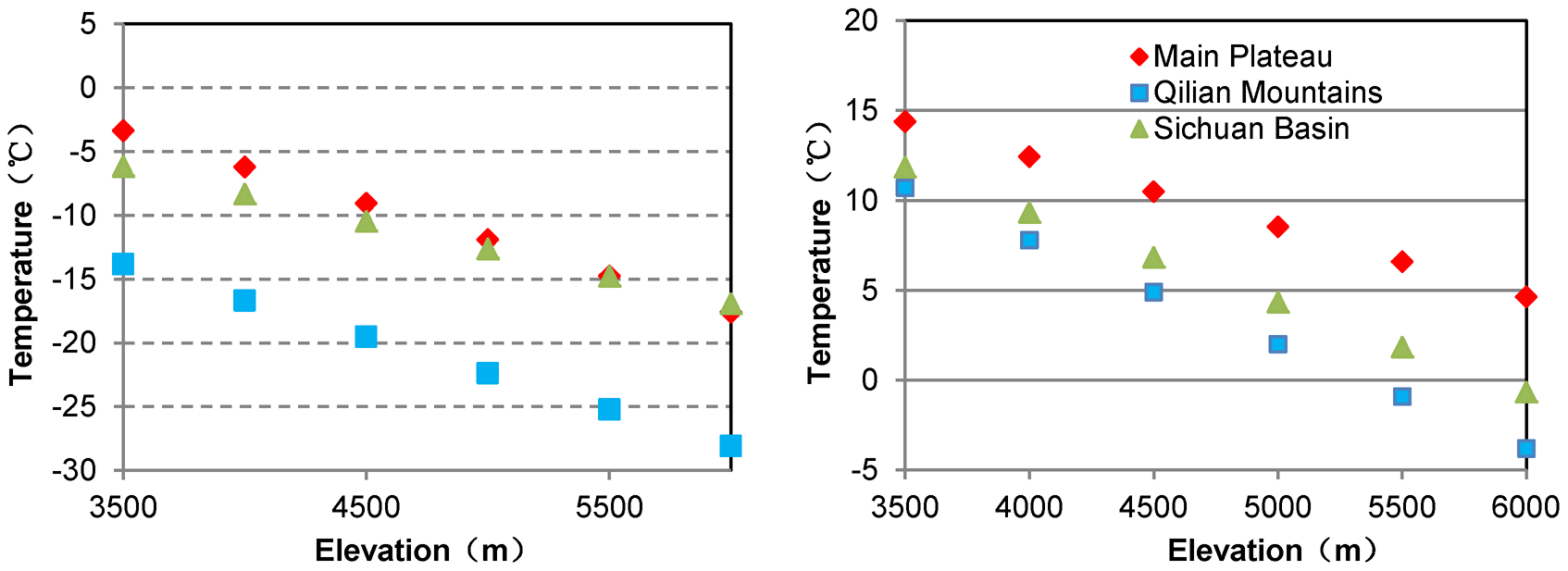
Temperatures with altitude for the three groups in January (left) and July (right).

According to the linear regression models of temperature with elevation (Table 2), temperatures at different elevations were calculated for the three regions. In the main Plateau, at altitudes of 4000–4500 m, there are 7 months (April through October) with a mean monthly temperature above 0°C; at altitudes of 4500–5000 m, there are 5 months (May through September) with a mean temperature above 0°C and 4 months (June through September) above 5°C; even at altitudes of 5000–6000 m, there are 4 months (June through September) with mean temperature above 0°C. For the warmest month (July), the mean temperature at altitudes 4000–4500 m can reach 10°C or higher (Table 3, Figure 3).

**Table 3:** Temperatures at altitudes 4000–6000 m in the main Plateau (unit: °C).

Elevation (m asl)	Jan.	Feb.	Mar.	Apr.	May	Jun.	Jul.	Aug.	Sept.	Oct.	Nov.	Dec.
4000	−6.2	−3.9	−0.4	3.6	7.2	10.9	12.4	12.1	9.7	4.0	−1.9	−5.2
4500	−9.1	−7.0	−3.5	0.7	4.6	8.8	10.5	10.1	7.7	1.4	−4.8	−7.9
5000	−11.9	−10.0	−6.5	−2.2	2.1	6.6	8.5	8.2	5.7	−1.1	−7.7	−10.7
5500	−14.8	−13.1	−9.6	−5.1	−0.5	4.5	6.6	6.2	3.7	−3.7	−10.6	−13.4
6000	−17.6	−16.1	−12.6	−8.0	−3.0	2.3	4.6	4.3	1.7	−6.2	−13.5	−16.2

In Qilian Mountains, at altitudes 4000–4500 m, there are 4–5 months (May through September) with monthly mean temperature above 0°C, and even in the warmest month (July), the temperature at this elevation cannot reach 10°C; at the altitudes 4500–5000 m, there are only 2–4 months (June through September) with mean temperature above 0°C and all of them are lower than 5°C; above 5000 m, there are almost lower than 0°C year-round (Table 4, Figure 3).

**Table 4:** Temperatures at altitudes 4000–6000 m in the Qilian Mountains (unit: °C).

Elevation (m a.s.l)	Jan.	Feb.	Mar.	Apr.	May	Jun.	Jul.	Aug.	Sept.	Oct.	Nov.	Dec.
4000	−16.68	−12.89	−8.29	−2.34	1.06	5.13	7.81	7.06	3.35	−3.30	−10.95	−15.80
4500	−19.53	−15.94	−11.54	−5.39	−2.14	2.03	4.91	4.16	0.70	−6.20	−14.10	−18.65
5000	−22.38	−18.99	−14.79	−8.44	−5.34	−1.07	2.01	1.26	−1.96	−9.10	−17.25	−21.50
5500	−25.23	−22.04	−18.04	−11.49	−8.54	−4.17	−0.90	−1.65	−4.61	−12.00	−20.40	−24.35
6000	−28.08	−25.09	−21.29	−14.54	−11.74	−7.27	−3.80	−4.55	−7.26	−14.90	−23.55	−27.20

In Sichuan Basin, at altitudes 4000–4500 m, there are 4–6 months (May through October) with mean temperature between 0°C–10°C; at the altitudes 4500–5000 m, there are only 4 months with temperature above 0°C and even in July, the highest mean temperature on the elevation 4500 m is 6.84°C; above 5000 m, the monthly mean temperature are going to lower than 0°C (except for in July and August) (Table 5 and Figure 3).

**Table 5:** Temperatures at altitudes 4000–6000 m in the Sichuan Basin (unit: °C).

Elevation (m a.s.l)	Jan.	Feb.	Mar.	Apr.	May	Jun.	Jul.	Aug.	Sept.	Oct.	Nov.	Dec.
4000	−8.33	−5.68	−3.61	−0.59	2.04	6.74	9.34	9.10	6.05	0.04	−4.12	−7.67
4500	−10.48	−7.93	−6.16	−3.39	−0.91	4.14	6.84	6.70	3.70	−2.56	−6.67	−9.92
5000	−12.63	−10.18	−8.71	−6.19	−3.86	1.54	4.34	4.30	1.35	−5.16	−9.22	−12.17
5500	−14.78	−12.43	−11.26	−8.99	−6.81	−1.06	1.84	1.90	−1.00	−7.76	−11.77	−14.42
6000	−16.93	−14.68	−13.81	−11.79	−9.76	−3.66	−0.66	−0.50	−3.35	−10.36	−14.32	−16.67

Moreover, on the same elevation, we can see, the monthly mean temperature in the main Plateau is higher than in the Sichuan Basin and the Qilian Mountains (Figure 3, Table 3,4 and 5), especially in the warm months. These results indicate that there is longer growing season and higher monthly mean temperature in the main Plateau than in its surroundings and adjacent lowlands. This shows that the main Plateau is really a tremendous heat source.

### Heating effect quantified by temperature difference between the main plateau, the Qilian Mountains and the Sichuan Basin

**Figure 4:**
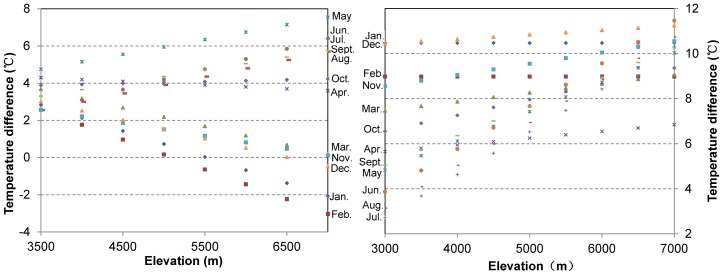
Temperature differences between main plateau and Sichuan Basin (left), and Qilian Mountains (right).

Comparison of air temperatures on same elevation with the Sichuan Basin and Qilian Mountains reveals that temperatures in the main plateau is higher than the free air on same elevation in the Qilian and Sichuan Basin (Figure 4, Table 6). From Tab. 6, we can see that, on elevation of 4500 m (the mean elevation of the plateau), temperature is evidently higher in the main plateau than in the Sichuan Basin. The difference is large (4–6°C) in warm months from April to October, and small (1–2°C) in other cold months. Moreover, these differences increase in warm months and decrease in winter (November through next year February) with higher altitudes (Figure 4). From individual observed data (Table 7), temperature of Lhasa station at 3649 m in July is 5.7°C higher than Leshan station in Sichuan Basin, and higher 5.6°C–9.3°C year-round. This means that the heating effect is strong in warm months and even evident in cold months.

**Table 6:** Temperatures and temperature differences (Δ*T*) in the three group areas at altitude 4500 m in the annual course (unit: °C).

Region	Jan.	Feb.	Mar.	Apr.	May	Jun.	Jul.	Aug.	Sept.	Oct.	Nov.	Dec.
Main plateau area	−9.1	−7.0	−3.5	0.7	4.6	8.8	10.5	10.1	7.7	1.4	−4.8	−7.9
Qilian Mountains	−19.5	−15.9	−11.5	−5.4	−2.1	2.0	4.9	4.2	0.7	−6.2	−14.1	−18.6
Sichuan Basin	−10.5	−7.9	−6.2	−3.4	−0.9	4.1	6.8	6.7	3.7	−2.6	−6.7	−9.9
Δ*T_Sichuan_*	1.4	1.0	2.7	4.1	5.5	4.6	3.6	3.4	4.0	4.0	1.9	2.0
Δ*T_Qilian_*	10.4	8.9	8	6.1	6.7	6.8	5.6	5.9	7.0	7.6	9.3	10.7

**Table 7:** Observed mean Temperatures, calculated mean temperatures at elevation of 3649 m and their differences between Lhasa station and Leshan station (unit: °C).

Station	LAT.	LONG.	ELEV.(m)	Jan.	Feb.	Mar.	Apr.	May	Jun.	Jul.	Aug.	Sept.	Oct.	Nov.	Dec.
Lhasa	29.67	91.13	3649	0.6	3.0	6.3	8.7	12.7	16.1	16.5	16.1	14.2	9.7	4.0	0.8
Leshan	29.57	103.75	424	7.4	10.7	14.3	19.0	22.4	24.4	26.9	26.0	22.8	18.3	14.2	8.7
Leshan*	29.57	103.75	3649	−12.0	−8.7	−5.0	−0.4	3.1	5.0	7.6	6.7	3.4	−1.0	−5.1	−10.6
Leshan#	29.57	103.75	3649	−6.5	−3.8	−2.1	0.9	3.4	7.6	10.8	10.5	7.6	1.6	−2.2	−5.8
ΔT1*	–	–	–	12.6	11.6	11.3	9.1	9.6	11.1	8.9	9.5	10.8	10.7	9.1	11.4
ΔT2#	–	–	–	7.1	6.8	8.4	7.8	9.3	8.5	5.7	5.6	6.6	8.1	6.2	6.5

Note: * and # are calculated temperature of Leshan at elevation 3649 m, the same elevation of Lhash station; and * Calculated by average lapse rate of 6.0°C km^−1^; # calculated by linear models in Table 2.

Air temperature of the main plateau is higher than in the Qilian Mountains year-round (Figure 4, Table 8). On elevation of 4500 m, the difference is small (5.9–7.0°C) in warm months, and large (6.1–10.7°C) in other cold months. Temperature differences increase with increased elevation year-round (Figures 4). Comparing Wudaoliang station in the northern Tibetan Plateau with Lintao station in Qilian Mountains, the former is 2.5–5.9°C higher than the latter year-round, and the difference is smaller in warm months than in cold months (Table 8). This shows that the main plateau is warmer than the surrounding mountains, and the heating effect is pronounced in the main plateau.

**Table 8:** Observed mean Temperatures, calculated mean temperatures at elevation of 3649 m and their differences between Wudaoliang station and Lintao station (unit: °C).

Station	LAT.	LONG.	ELEV.(m)	Jan.	Feb.	Mar.	Apr.	May	Jun.	Jul.	Aug.	Sept.	Oct.	Nov.	Dec.
Wudaoliang	35.22	93.08	4612.20	−15.3	−13.0	−9.6	−4.5	−0.8	3.1	6.7	6.2	2.7	−4.3	−11.0	−14.1
Lintao	35.35	103.85	1893.80	−5.7	−0.7	4.2	9.5	13.7	17.1	19.3	18.7	13.8	8.3	1.7	−4.5
Lintao*	35.35	103.85	4612.20	−22.0	−17.0	−12.1	−6.8	−2.6	0.8	3.0	2.4	−2.5	−8.0	−14.6	−20.8
Lintao#	35.35	103.85	4612.20	−21.2	−17.3	−13.5	−7.1	−3.7	0.2	3.6	2.9	−0.6	−7.5	−15.4	−20.0
ΔT1*	–	–	–	6.7	4.0	2.6	2.3	1.8	2.3	3.7	3.8	5.2	3.7	3.6	6.7
ΔT2#	–	–	–	5.9	4.3	3.9	2.5	2.9	2.9	3.2	3.2	3.3	3.2	4.4	5.9

Note: * and # are calculated temperature of Lintao at elevation 4612.2 m, the same elevation of Wudaoliang station, and * Calculated by average lapse rate of 6.0°C km^−1^; # calculated by linear models in Table 2.

### Heating effect makes the occurrence of the highest timberline of the northern hemisphere in the southeastern Tibetan Plateau

Above analysis shows that the temperatures at high altitudes in main plateau are warmer than adjacent free air in surrounding areas due to the heating effect. On elevation 4500 m, the growing season can up to 4–5 months (mean temperature above 5°C), and in the warmest month (July), the monthly mean temperature can reach 10.5°C (Table 3). This provides temperature condition for forest development in such high elevation, for the 10°C isotherm of the warmest month was considered to be coincided with the alpine timberline [3]. According to our analysis, the 10°C isotherm of the warmest month lies between 4600–4700 m, which strictly confirms the result of previous timberline research of the plateau [2]–[4].

Besides above calculated temperatures with altitudes, observed temperatures in the main Plateau also confirm that it is possible for mean temperature higher than 10°C at altitudes 4600–4700 m in July. For example, observed mean temperature of Lhasa station (located at: 29.67°N, 91.13°E, in southern main Plateau; Elevation: 3649 m) in July is 16.5°C, which is close to mean temperatures of Wuhan and Changsha in April, then even calculating by average lapse rate of 6.0°C km^−1^, the mean temperature of mountains near Lhasa station at altitude 4649 m can reach 10.5°C (Table 7). In fact, the temperature lapse rate in the main Plateau may be lower than 6.0°C km^−1^ in summer according to related researches (Hastenrath (1968), Forster (1982) and Flenley (2007) have stated that lapse rates were somewhat steeper over lowlands than over large mountain masses [10], [29], [30]). It means that mountain temperatures in the main Plateau at altitudes 4649 m can reach 10.5°C or higher. On a global or continental scale, temperature is the final impact factor to determine timberline altitude [31]. Our results show that the 10°C isotherm of the warmest month lies between 4600–4700 m in the main Plateau, the highest 10°C isotherm of the warmest month in the northern hemisphere. This may help explain why the highest timberline in the northern hemisphere occurs on the Tibetan Plateau.

## Discussion and Conclusions

### Discussion

#### Temperature lapse rate

Normally when we study mountain climate, temperature lapse rate is a necessary parameter [32]. In this paper, average lapse rate of −0.60°C/100 m is used to adjust the temperature of observed stations (Table 7 and 8). However, such average value is known to be rough approximations unsuitable in more precise studies [33]–[34], though average temperature lapse rates of −0.55°/100 m [35], −0.60°C/100 m [36], or −0.65°C/100 m [37] are often used when low precision suffices [32]. Related researches revealed that the lapse rate at which air cools with elevation varies from about −0.98°C/100 m for dry air (the dry-air adiabatic lapse rate) to about −0.48°C/100 m (the saturated adiabatic lapse rate [36]). Dry adiabatic lapse rate is very different than wet. In Tibetan Plateau, the humidity is quite different in places and in seasons [38]–[39], in turns the lapse rate in the Plateau will vary widely in different places and months. Moreover, lapse rates for the Tibetan Plateau may be smaller than the average temperature lapse rate given the heating effect of the Tibetan Plateau, because temperature lapse rates are steeper on isolated mountains near the sea than on extensive mountain ranges that provide their own heating [30], [40], and the greater the heating effect of the mountains, the smaller the average lapse rates. It is also noted that lapse rates show considerable variability in relation to climatic zone, season [30], air mass type [41], and local topography [10], [23]. So it will be important to study the lapse rate for the Tibetan Plateau in future.

#### Why does the highest timberline of the northern hemisphere occur in the southeastern Tibetan Plateau, but not in other places of the Tibetan Plateau?

Our results shows that the 10°C isotherm of the warmest month can lie between 4600–4700 m in the Tibetan Plateau, and this is the necessary temperature condition for forest growth. However, that does not mean all 4600–4700 m mountain areas are suitable for forest development. It may higher than 10°C in south Tibetan Plateau but lower than 10°C in north Tibetan Plateau at 4600–4700 m, for example the Lhasa station and the Wudaoliang Station (Table 4 and Table 5). Besides temperature, precipitation is also an important factor for forest growth [42]–[43]. In Tibetan Plateau, the annual mean precipitation decreases from 2,000–2,500 mm in the southeast to about 50–100 mm in the northwest [4], [38], [44], and the aridity increases from less than 1 to 25 [39]. The cold and dry climate in northwestern Plateau is not suitable for forest development. Usually, forest could only develop in regions with annual rainfall of over 500 mm. That is why the highest timberline occurs in southeastern Tibetan Plateau.

### Conclusions

(1) The heating effect of the Tibetan Plateau is significant. The mean monthly temperature, on the elevation of 4500 m, is higher in the main plateau than in the neighboring regions, usually 1–6°C higher than over the Sichuan Basin and 5.9–10.7°C higher than in the Qilian Mountains in the northeastern plateau. (2) At altitudes of 4000–5000 m on the main plateau, there are 5–7 months with a mean temperature above 0°C, and 4–5 months above 5°C. Even at altitudes of 5000–6000 m, there are 4 months with a mean temperature above 0°C. The mean temperature of the warmest month (July) can reach 10°C at about 4600–4700 m, providing favorable temperature condition for forest growth at this high elevation.
